# Ameliorative Effects of *Tribulus Terrestris* (Qutayb) Extracts Against Gentamicin-Induced Nephrotoxicity in Wistar Rats

**DOI:** 10.1155/ijne/7620700

**Published:** 2025-10-29

**Authors:** Ahmed Al-Mohamadi, Imadeldin M. Taj Eldin, Doa'a Ibrahim, Rowaida Albadani

**Affiliations:** ^1^Department of Clinical Pharmacy and Pharmacy Practice, Faculty of Pharmacy, University of Science and Technology, Sana'a, Yemen; ^2^Department of Pharmacology, Faculty of Pharmacy, Omdurman Islamic University, Khartoum, Sudan; ^3^Department of Pharmacology, Faculty of Pharmacy, Al-Razi University, Sana'a, Yemen; ^4^Department of Pharmacology, Faculty of Pharmacy, University of Gezira, Medani, Sudan

**Keywords:** acetonitrile, aminoglycosides, antioxidant, different polarities, ethanol, nephrotoxicity, oxidative stress

## Abstract

*Tribulus terrestris* has a long-standing historical legacy, having been used for centuries in the treatment of various kidney disorders and other health issues. This research aimed to evaluate the kidney-protective and antioxidant effects of different *Tribulus terrestris* extracts in ameliorating gentamicin-induced nephrotoxicity in Wistar albino rats. The study included nine rat groups of Wistar rats (*n* = 6 per group) treated for 14 days. The control group was given intraperitoneal injections of normal saline and dimethyl sulfoxide (DMSO). Group 2 received subcutaneous gentamicin injections at a dose of 100 mg/kg for eight days. Groups 3–5 were given an intraperitoneal ethanol extract of *Tribulus terrestris* (EETT) at 150, 300, and 450 mg/kg, respectively. Groups 6–8 received an intraperitoneal acetonitrile extract of *Tribulus terrestris* (AETT) at 150, 300, and 450 mg/kg, respectively. Group 9 was administered 200 mg/kg of intraperitoneal N-acetylcysteine. The results revealed that the EETT exhibited a protective effect against gentamicin-induced renal damage in Wistar albino rats. This protection was shown by reduced kidney tissue damage, lower MDA levels, and increased antioxidant enzyme levels, CAT, and GSH. Moreover, EETT administration dose-dependently improved renal histology, with full recovery observed at 450 mg/kg, whereas AETT showed no protective effect suggesting a potential dose-dependent effect. Additional studies are necessary to investigate how EETT's dose–response curve affects its ability to treat kidney damage induced by gentamicin in rats. Conversely, various doses of AETT failed to show a protective effect against gentamicin-induced nephrotoxicity.

## 1. Introduction

Kidney disorders are among the leading causes of morbidity and mortality worldwide and thus represent a critical public health concern. These conditions encompass a broad spectrum of clinical entities—including acute kidney injury (AKI), chronic kidney disease (CKD), nephrolithiasis, and urinary tract infections (UTIs). Despite differences in etiology and clinical presentation, many renal diseases share core pathogenic pathways—chiefly oxidative stress, inflammation, and progressive nephron loss—that ultimately impair kidney function [1]. To evaluate nephroprotective strategies, gentamicin (GNT)-induced AKI in rodents serves as a well-established experimental model.

GNT, a widely used aminoglycoside antibiotic for severe gram-negative infections, is commonly employed to induce experimental AKI due to its well-characterized nephrotoxic profile [2]. GNT-induced nephrotoxicity is commonly evidenced by elevated serum levels of creatinine, urea, and uric acid, in addition to increased renal malondialdehyde (MDA) and decreased glutathione (GSH) content. These biochemical disturbances are typically accompanied by histopathological alterations, including tubular necrosis and glomerular damage, and are widely employed as experimental indicators of oxidative stress and renal dysfunction in animal models [3].

The main mechanisms underlying GNT-induced nephrotoxicity are oxidative stress, inflammation, and mitochondrial dysfunction. Upon accumulation in renal proximal tubular epithelial cells, GNT forms iron–drug complexes and induces the activation of inducible nitric oxide synthase (iNOS), resulting in excessive production of reactive oxygen and nitrogen species (ROS/RNS). These reactive intermediates contribute to lipid peroxidation, DNA fragmentation, disruption of cellular membranes, inhibition of catalase (CAT) activity, and depletion of endogenous antioxidants, such as GSH. In addition, GNT has been shown to trigger apoptotic pathways and to ameliorating tissue injury characterized by tubular and glomerular degeneration [4–7].

N-Acetylcysteine (NAC) is a thiol-containing antioxidant that serves as both a precursor to GSH and a direct scavenger of ROS. It has demonstrated protective effects in various models of drug-induced nephrotoxicity, including GNT, mainly by restoring intracellular redox balance and ameliorating oxidative tissue injury [8].

*Tribulus terrestris* (*T. terrestris*) from the Zygophyllaceae family is a valuable herb renowned for its traditional medicinal use across various regions. It is rich in bioactive compounds like flavonoids, saponins, alkaloids, phytosterols, glycosides, and other various ingredients [9]. Plant-derived natural chemicals have long been focusing of research as potential therapeutic agents for a variety of health issues [10]. According to previous studies, *T. terrestris* was traditionally used as an antihypertensive, antiurolithiasis, diuretic, analgesic, antihyperlipidemic, immune-modulating, hypoglycemic, anticancer, antihelminthic, aphrodisiac, hepatoprotective, anti-inflammatory [11–13], and antioxidant [14]. Various studies have examined the effects of *T. terrestris* on various disorders, including kidney disease, anticancer, palliative, tonic, hypertension, urolithiasis, Parkinsonism, and urinary infection. Important findings reveal that *T. terrestris* extract demonstrated antioxidative properties, vasodilatory effects, aphrodisiac, cooling, diuretic, tonic, and antiapoptotic properties [15, 16]. On the other hand, an Iranian study highlighted a case of *T. terrestris–induced* nephrotoxicity and hepatotoxicity in a male individual, despite the traditional belief that *T. terrestris* aids in the prevention of kidney stone formation [17].

*Tribulus terrestris* has been traditionally used for centuries in various medical systems, including Ayurveda, Traditional Chinese Medicine, and folk practices in regions such as India, South Africa, Turkey, and Bulgaria, particularly for managing kidney and urinary tract conditions [11]. Similarly, in Yemen, patients frequently use herbal remedies as a substitute for or in addition to their prescribed treatments for minor health issues before turning to allopathic medicines [18]. However, the safety and efficacy of numerous medicinal plants remain inadequately researched. *Tribulus terrestris* is commonly used in Yemeni traditional medicine, especially in rural regions, to address various kidney problems. The objective of this study is to evaluate the renal protective and antioxidant properties of *T. terrestris* extracts on GNT-induced nephrotoxicity in Wistar albino rats.

## 2. Materials and Methods

### 2.1. Plant Collection and Identification

The entire *T. terrestris* plant, including its leaves, stems, roots, and fruits, was collected during May and June 2023 from selected natural habitats in the governorates of Taiz and Ibb in Yemen's central highlands at an estimated elevation between 1800 and 2000 m above sea level. Plant species were accurately identified by the specialist in the Botany Department at the Faculty of Science, Sanaa University, Yemen. A voucher specimen (No. 721) was preserved at the Yemeni National Herbarium, Sana'a University, Yemen.

### 2.2. Chemicals and Medicines

Sigma-Aldrich's local distributor supplied analytical grade solvents (ethanol, acetonitrile, n-hexane, and dimethyl sulfoxide [DMSO]). GNT ampule (80 mg/2 mL; Gentamicin) in the clinical formulation was procured from Krka, dd (Novo Mesto, Slovenia). NAC 300 mg/3 mL (Asist) was obtained from Bilim (Istanbul, Turkey). MDA, CAT, and GSH were bought from Biodiagnostic (Cairo, Egypt).

### 2.3. Extraction Procedure

The collected plant was cleaned process with clean water to eliminate any dust and dirt from its parts. Subsequently, the material was then dried in the shade and finely ground using a motorized blender. Three sequential extraction methods using solvents of increasing polarity were employed to achieve comprehensive extraction of both polar and nonpolar compounds: n-hexane (low polarity), acetonitrile (medium polarity), and ethanol (high polarity). The extraction method followed the procedures described by Sasidharan et al. with some modification [19, 20]. The plant powder (100 g) was placed in a glass jar and soaked in the solvent. The jar was sealed with aluminum foil and left for 48 h, with periodic shaking and stirring on a vibrating shaker (RS 10 BASIC YELLOWLINE) at 120 rpm. The extraction was repeated twice to ensure complete extraction. The organic extracts were filtered through Whatman filter paper No. 1, followed by the evaporation of individual solvents under vacuum using a Buchi R200 Rotary evaporator. A Labconco freeze-dryer was then used for the drying process.

### 2.4. Sample Preparation

The solid residue extracts were dissolved separately in a solution of DMSO and normal saline and sterilized through 0.45-μm Whatman filter papers. The final DMSO concentration was kept at 4% during assays. The n-hexane extract was excluded from the study due to its low yield, which was insufficient for both the phytochemical and biological evaluations.

### 2.5. Phytochemical Screening

Qualitative phytochemical screening of the EETT and AETT was carried out following the standard procedures described by Sofowora (1993), Trease and Evans (2002), and Harborne (1973) [21–23]. The screening included a foam test for saponins, Dragendorff's and Mayer's reagents for alkaloids, aluminum chloride and ammonia tests for flavonoids, Borntrager's test for anthraquinones, ferric chloride test for tannins, Salkowski test for terpenoids, and resin test using acetic anhydride and sulfuric acid.

### 2.6. Experimental Animals

Normal, mature male Wistar rats, weighing 180 ± 20 g, were obtained from the University of Science and Technology's animal facility in Sana'a, Yemen. The rats were acclimated in groups of six per cage under regulated environmental conditions, including a temperature of 23 ± 2°C and alternating 12 h light and dark cycles. The animals had ad libitum access to food and water. According to the National Research Council's Animal Care and Use Committee's rules, all experimental procedures were carried out during the light cycle. Throughout the investigation, efforts were undertaken to limit the number of animals involved and alleviate their distress. Ethical approval (no. EAC/UST233) was obtained from the Ethical Committee of the University of Science and Technology prior to the commencement of the experiments. The rats were acclimated to the laboratory environment for 2 weeks prior to the start of the trials.

### 2.7. Study Design

In an experimental study, healthy and mature male Wistar rats (*N* = 54) were randomly distributed among nine groups, each comprising six rats. The experimental groups were as follows:

 
*Group 1 (Negative Control)*: During a 14-day period, the rats in this group received intraperitoneal (IP) injections of normal saline and DMSO.
 
*Group 2 (Positive control: GNT-induced nephrotoxicity)*: This group received a subcutaneous injection of GNT at a dose of 100 mg/kg for eight consecutive days in the neck region, following the methodology of Walker and Shah [24].
 
*Groups 3–5: treated with ethanol extract of T. terrestris (EETT)*: Three different dose treatment groups as follows: GNT/EETT-150, GNT/EETT-300, and GNT/EETT-450.
  These groups received subcutaneous GNT injections (100 mg/kg) in the neck region for eight days, along with IP injections of EETT at doses of 150, 300, or 450 mg/kg from day one to Day 14 of the study. Dosages were determined based on Chaudhary et al. [25], with modifications informed by a pilot trial that was set up to assess the nature of the drug.
 
*Groups 6–8: treated with acetonitrile extract of T. terrestris (AETT)*: Three different dose treatment groups as follows: GNT/AETT-150, GNT/AETT-300, and GNT/AETT-450.
  These groups followed a similar protocol to the EETT groups, receiving subcutaneous GNT injections (100 mg/kg) in the neck area for eight days, combined with IP injections of AETT at doses of 150, 300, or 450 mg/kg throughout the 14-day study period.
 
*Group 9: treated with standard drug*: This group received subcutaneous GNT injections (100 mg/kg) in the neck region for eight days, along with IP injections of NAC at a dose of 200 mg/kg throughout the 14-day study period [26].

On the 15th day of the experiment, 24 h after their final feeding, the rats were euthanized using diethyl ether anesthesia. Blood samples were collected via venipuncture for kidney biomarker analysis. Subsequently, both kidneys were carefully removed for histopathological examination.

### 2.8. Biochemical Parameters

Kidney function tests, including serum creatinine, blood urea nitrogen (BUN), and uric acid levels, were assessed using a COBAS 6000 analyzer.

### 2.9. Histopathological Examination

The histopathological examination of the kidney was carried out by initially fixing the kidney in 10% neutral buffered formalin for 24 h. Subsequently, the samples were washed in normal saline, dehydrated in alcohol, cleared in xylene, and embedded in paraffin wax. Sections of 5 μ thickness were cut, stained with hematoxylin and eosin, following Bancroft and Gamble's guidelines [27], and examined under a light microscope (Leica Microsystems, Germany) at a magnification of × 400. Histopathological evaluation was performed by a histopathologist in a blinded manner. The assessment was based on qualitative comparison of key histological features, such as tubular necrosis, medullary congestion, and proteinaceous casts, among experimental groups [28].

### 2.10. Assessment of Antioxidant Activity in Renal Tissue

The rat kidneys were homogenized in ice-cold 10% trichloroacetic acid phosphate-buffered saline with heparin to remove blood cells and clots. After centrifugation, MDA, CAT, and GSH levels were measured in the supernatant. The process described by Hamed et al. was applied in order to prepare the MPs stock solution [29]. CAT, GSH, and MDA levels were assessed using a colorimetric method with commercial kits following the manufacturer's instructions. MDA levels were determined by reacting MDA with thiobarbituric acid (TBA) under specific conditions, with absorbance measured at 534 nm following the method described by Ohkawa et al. [30]. The GSH content in tissue homogenates was assessed following the procedure outlined by Carlberg and Mannervik [31], which involves the reaction of Ellman's reagent with GSH to produce a yellow-colored product, with absorbance measured at 412 nm. CAT activity was determined using the Aebi method [32] to measure hydrogen peroxide breakdown at 240 nm.

### 2.11. Statistical Analyses

Data were analyzed using GraphPad Prism Version 8.0 and are expressed as mean ± standard error of the mean (SEM). One-way ANOVA followed by Tukey's post-test was used to compare biomarkers among the research groups. Statistical significance was set at a *p* value < 0.05.

## 3. Results

### 3.1. Extracts Yield

The yields of the extracts were 5.7%, 2%, and 0.3% for ethanol, acetonitrile, and n-hexane, respectively.

### 3.2. Phytochemical Screening

Saponins, alkaloids, flavonoids, anthraquinones, and tannins were identified in EETT screening. In contrast, the AETT detected only alkaloids, terpenoids, and resins (Table 1).

### 3.3. Main Experiment Results

#### 3.3.1. The Impact of EETT and AETT and/or GNT on Serum Creatinine Levels in Rats

**Figure 1:**
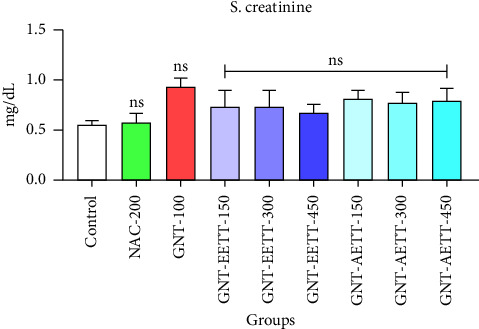
Impact of ethanol and acetonitrile extracts of *Tribulus terrestris* and/or gentamicin on serum creatinine levels in rats. Each bar is expressed as mean ± SEM. GNT vs. control. GNT vs. GNT/NAC. GNT vs. (GNT/EETT: 150, 300, and 450 mg/kg). GNT vs. (GNT/AETT: 150, 300, and 450 mg/kg); no: nonsignificant; *n* = 6 rats/group.

Subcutaneous GNT injection (100 mg/kg) for eight days led to an increase in serum creatinine levels, though not statistically significant (*p* < 0.069; Figure 1) compared to the control group. IP administration of EETT at various doses demonstrated an ameliorative effect. However, this improvement was not statistically significant when compared to the GNT-100 group (Figure 1). Similarly, varying AETT doses did not significantly reduce serum creatinine levels (*p* < 0.05; Figure 1) compared to the GNT-100 group.

#### 3.3.2. The Impact of EETT and AETT and/or GNT on Serum Urea Levels in Rats

**Figure 2:**
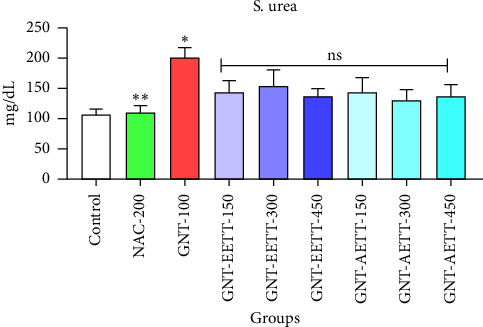
Impact of ethanol and acetonitrile extracts of *Tribulus terrestris* and/or gentamicin on serum urea levels in rats. Each bar is expressed as mean ± SEM. GNT vs. control: ^∗^*p*=0.0025; GNT vs. GNT/NAC: ^∗∗^*p*=0.0031; GNT vs. GNT/EETT: 150, 300, and 450 mg/kg. GNT vs. GNT/AETT: 150, 300, and 450 mg/kg; no: nonsignificant; *n* = 6 rats/group.

Eight-day subcutaneous GNT treatment significantly elevated serum urea levels (*p* < 0.0025; Figure 2) compared to the control group. IP administration of EETT at a dose of 450 mg/kg resulted in a marked decrease (31.6%) in blood urea levels. However, there was no statistically significant reduction observed at any dose of either EETT or AETT compared to the GNT group.

#### 3.3.3. The Impact of EETT and AETT and/or GNT on Serum Uric Acid Levels in Rats

**Figure 3:**
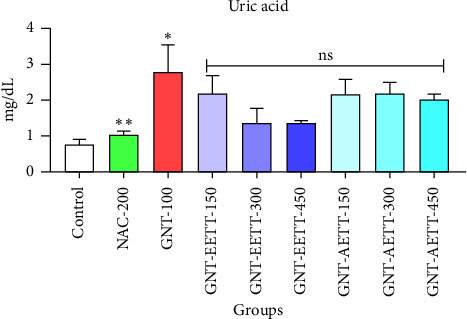
Impact of ethanol and acetonitrile extracts of *Tribulus terrestris* and/or gentamicin on serum uric acid levels in rats. Each bar is expressed as mean ± SEM. GNT vs. control: ^∗^*p*=0.0033; GNT vs. GNT/NAC: ^∗∗^*p*=0.0106; GNT vs. GNT/EETT: 150, 300, and 450 mg/kg. GNT vs. GNT/AETT: 150, 300, and 450 mg/kg; no: nonsignificant; *n* = 6 rats/group.

Uric acid levels were assessed to evaluate EETT and AETT effects. Subcutaneous administration of GNT resulted in a significant increase (*p* < 0.05) in uric acid levels compared to the control group (Figure 3). IP administration of EETT at 300 and 450 mg/kg doses showed a marked decrease (50% and 52.4%, respectively) in serum uric acid levels. However, these reductions were not statistically significant (*p* < 0.070 and 0.052; Figure 3) compared to the GNT group. IP administration of 150 mg/kg EETT and various doses of AETT had no significant impact on blood uric acid levels.

#### 3.3.4. The Results of the Antioxidant Examination

**Figure 4:**
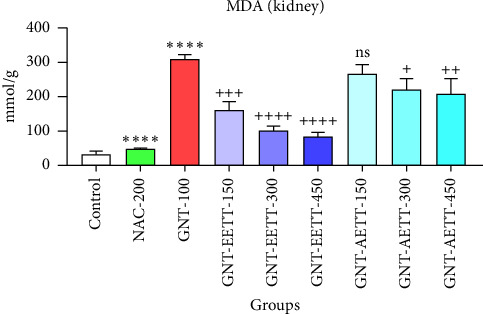
Impact of ethanol and acetonitrile extracts of *Tribulus terrestris* and/or gentamicin on malondialdehyde (MDA). Each bar is expressed as mean ± SEM. GNT vs. control. GNT vs. GNT/NAC: ^∗∗∗∗^*p* < 0.0001; GNT vs. GNT/EETT: 150 mg/kg: ^+++^*p*=0.0005; GNT vs. GNT/EETT: 300 and 450 mg/kg: ^++++^*p* < 0.0001; GNT vs. GNT/AETT: 150 mg/kg: no: nonsignificant; GNT vs. GNT/AETT: 300 mg/kg: ^+^*p*=0.049; GNT vs. GNT/AETT: 450 mg/kg: ^++^*p*=0.0289; *n* = 6 rats/group.

**Figure 5:**
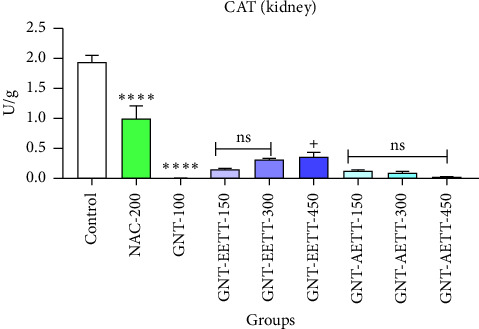
Impact of ethanol and acetonitrile extracts of *Tribulus terrestris* and/or gentamicin on antioxidant enzyme CAT. Each bar is expressed as mean ± SEM. GNT vs. control. GNT vs. GNT/NAC: ^∗∗∗∗^*p* < 0.0001; GNT vs. GNT/EETT: 450 mg/kg: ^+^*p*=0.0362; GNT vs. GNT/EETT: 150 and 300 mg/kg. GNT vs. GNT/AETT: 150, 300, and 450 mg/kg; no: nonsignificant; *n* = 6 rats/group.

**Figure 6:**
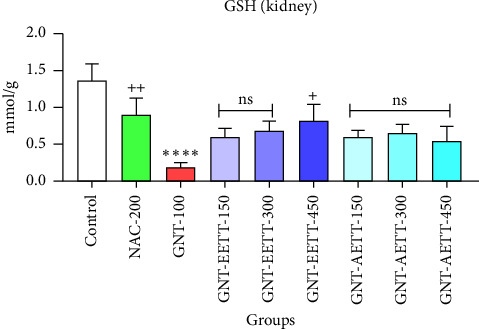
Impact of ethanol and acetonitrile extracts of *Tribulus terrestris* and/or gentamicin on antioxidant enzyme GSH. Each bar is expressed as mean ± SEM. GNT vs. control. ^∗∗∗∗^*p* < 0.0001; GNT vs. GNT/NAC: ^++^*p*=0.0124; GNT vs. GNT/EETT: 450 mg/kg: ^+^*p*=0.0392; GNT vs. GNT/EETT: 150 and 300 mg/kg. GNT vs. GNT/AETT: 150, 300, and 450 mg/kg; no: nonsignificant; *n* = 6 rats/group.

GNT administration at 100 mg/kg significantly (*p* ≤ 0.0001) increased the MDA levels in kidney tissue, while also significantly (*p* ≤ 0.0001) reducing the levels of cellular antioxidant enzymes such as GSH and CAT compared to the control group (Figures 4, 5, and 6). However, different doses of EETT exhibited a significant decrease in MDA levels in kidney tissue at a dose of 150 mg/kg (*p*=0.0005), 300 mg/kg, and 450 mg/kg (*p* ≤ 0.0001) compared to the GNT group, as shown in Figure 4. Additionally, the high dose (450 mg/kg) of EETT significantly increased the antioxidant enzymes GSH and CAT levels (Figures 5 and 6). On the other hand, various doses of AETT demonstrated a significant (*p* ≤ 0.05) reduction in MDA levels at only moderate and high doses (300 mg/kg and 450 mg/kg). Interestingly, AETT showed no significant effect on the levels of GSH and CAT enzymes, as shown in Figures 5 and 6.

#### 3.3.5. The Histopathological Examination

**Figure 7:**
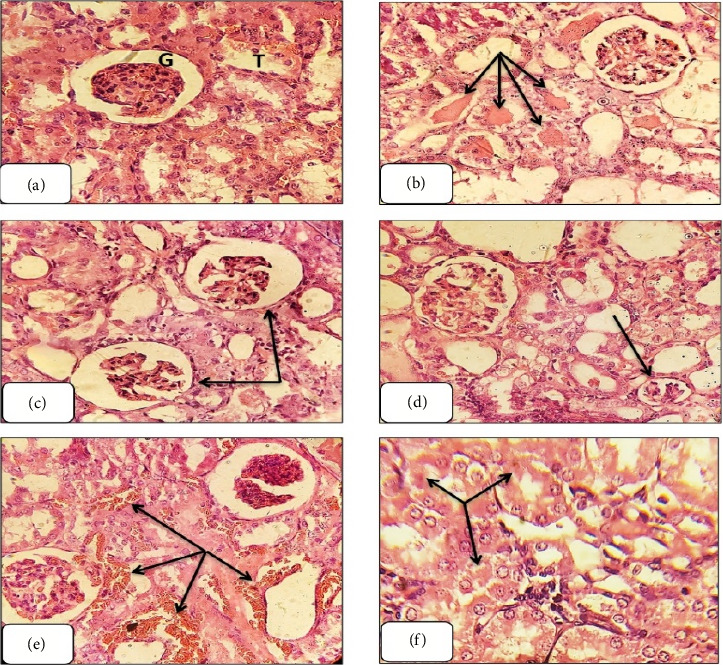
Rat kidney sections from different groups. (a): rat kidney from control group shows normal architecture of glomerulus (G) and normal tubules (T). The rest are rat kidney sections treated with GNT-100 showing different nephrotoxicity like tubular casts (b), glomerular degeneration (c), glomerular shrinkage (d), kidney hemorrhage (e), and tubular necrosis (f). (400X, H&E).

Histological examination of hematoxylin and eosin-stained kidney sections from GNT-treated rats revealed significant structural changes, including tubular casts, glomerular shrinkage and degeneration, kidney hemorrhaging, and tubular necrosis (Figures 7(a), 7(b), 7(c), 7(d), 7(e), and 7(f)).

**Figure 8:**
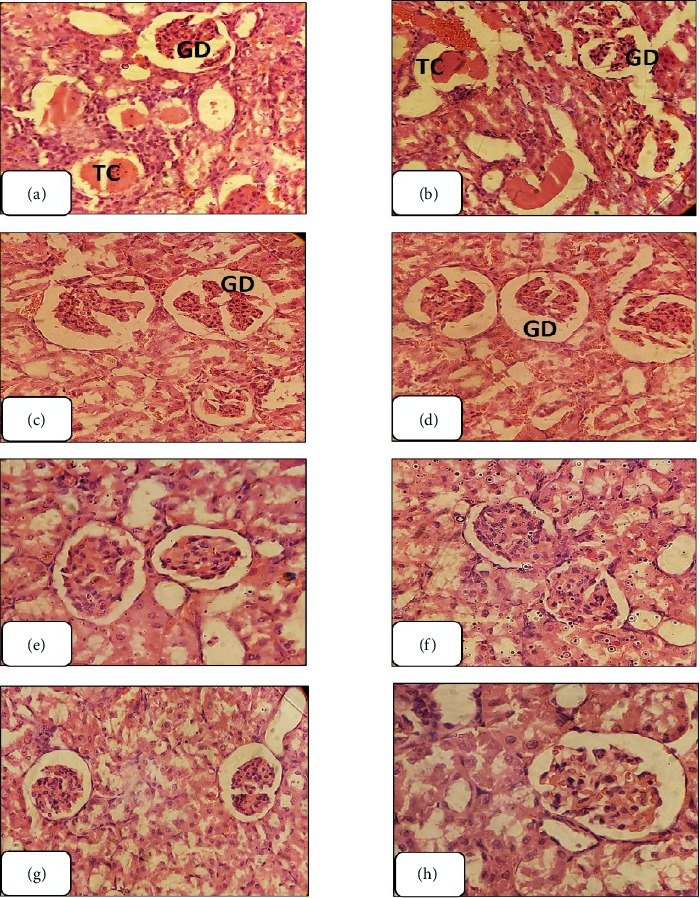
Rat kidney sections from different treatment groups. (a and b): rat kidney sections treated with ETTE (150 mg/kg) show healing from nephrotoxicity except glomerular degeneration (GD) and tubular cast (TC). (c and d): rat kidney sections treated with plant extract (300 mg/kg) show more healing from nephrotoxicity except glomerular degeneration (GD). (e and f): rat kidney sections treated with plant extract (450 mg/kg) show total healing from nephrotoxicity. (g and h): rat kidney sections treated with standard drug show approximately total healing from nephrotoxicity. (400X, H&E).

**Figure 9:**
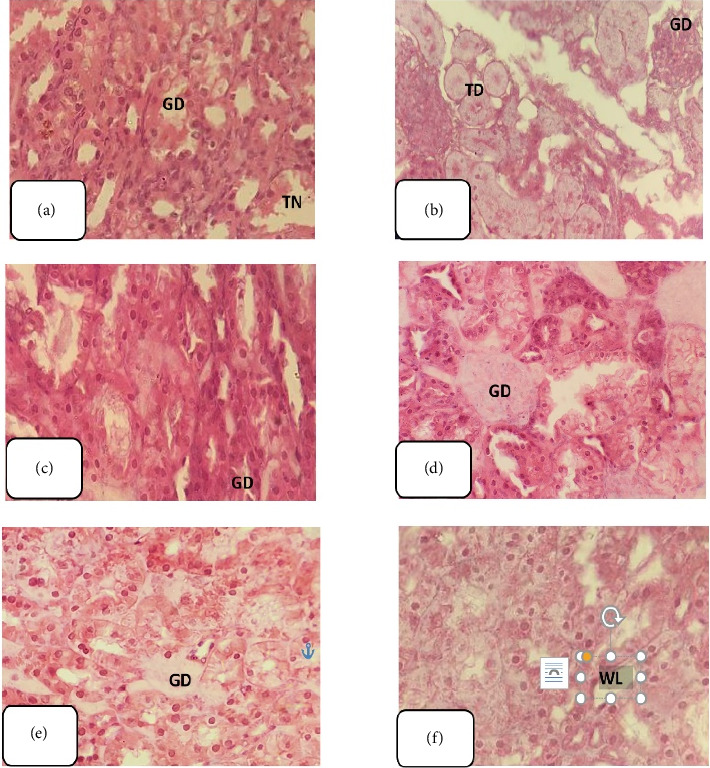
Rat kidney sections from different ATTE treatment groups. Rat kidney sections treated with acetonitrile plant extract (150, 300, and 450 mg/kg) show no healing from nephrotoxicity. (a–f) glomerular degeneration (GD) and tubular necrosis (TN), cellular swelling and tubular dilatation (TD), and wide lumina (L), and the parenchymal alterations contained of degeneration of the tubules and the interstitial tissues infiltrated with eosinophils and lymphocytes. (400X, H&E).

The administration of EETT at a dose of 150 mg/kg resulted in restoration of renal tissue integrity with the exception of glomerular degeneration and tubular casts. Kidney sections from rats treated with EETT at a dose of 300 mg/kg exhibited significant amelioration of nephrotoxicity, except for glomerular degeneration. Treatment with EETT at 450 mg/kg revealed complete recovery from nephrotoxicity, similar to the kidney sections treated with the standard drug, NAC, demonstrating nearly full recovery (Figure 8). Conversely, kidney sections treated with AETT at various doses (150, 300, and 450 mg/kg) did not exhibit any improvement from GNT-induced renal toxicity (Figure 9).

## 4. Discussion

The kidneys are essential for controlling blood volume and eliminating hazardous chemicals from the body; they are susceptible to toxicity and damage from medication, because of their high capacity for drug absorption [33]. Various drugs have been associated with nephrotoxicity when used therapeutically [34]. GNT, a commonly prescribed aminoglycoside antibiotic, is well known for its significant contribution to drug-induced renal injury [7]. The purpose of this study is to investigate the nephroprotective effects of different *Tribulus terrestris* (*T. terrestris*) extracts in rats with GNT-induced AKI. The results of this study advance our knowledge of phytochemicals in renal protection by offering important new information about the potential therapeutic benefits of EETT and AETT.

According to the results of this study, administering GNT subcutaneously for eight days induced renal damage, as evidenced by elevated levels of urea, and uric acid. Additionally, GNT-induced oxidative stress in kidney tissue led to an increase in the levels of an indicator of lipid peroxidation, MDA, and a reduction in cellular antioxidant enzymes, including CAT and GSH. Furthermore, significant renal histological alterations were also observed, including tubular casts, glomerular degeneration and shrinkage, kidney hemorrhage, and tubular necrosis. These results are in line with earlier research [10, 35–37]. Previous studies have shown various ways in which GNT causes kidney damage. The accumulation of GNT in the kidney's outer layer leads to changes in kidney structure, with notable parallels observed between human and animal studies. GNT acts as a chelating agent for iron, extracting it from mitochondria in the renal cortex and creating iron–GNT complexes, and these complexes promote ROS/RNS generation [6].

Studies have reported that GNT activation of the inducible form of nitric oxide synthase in renal tissue increases the synthesis of O_2_ and nitric oxide. Inhibiting these enzymes can alleviate kidney injury induced by GNT-triggered oxidative stress [5]. The excessive generation of ROS and RNS damages cells by disrupting their proteins, lipids, and DNA [35, 38]. Furthermore, GNT-induced nephrotoxicity results in reduced renal levels of GSH and GST, as demonstrated by Manikandan et al. [39], and this reduction according to Gutteridge [40] enables the interaction of ROS with cellular constituents, such as lipids, proteins, carbohydrates, and nucleic acids, which eventually causes damage to renal tissue.

The protective effect of a greater dose (450 mg/kg) of EETT on cells in this experimental study was observed through increased activity of antioxidant enzymes, such as GSH and CAT, reduced oxidative stress, and inhibition of free radical generation by lowering MDA. The potential of EETT to ameliorate oxidative stress has been associated with a higher total phenolic content in EETT, as revealed through phytochemical analysis of this investigation, which aligns with Reshma et al. [41], who suggested that *T. terrestris's* protective effects were possibly due to a number of chemical components, such as flavonoids, alkaloids, and saponins. Flavonoids and saponins are thought to be the most common phytoconstituents. These findings are supported by prior research studies [42–44].

Based on the outcomes of this study, GNT injection in rats induced significant renal tissue damage, including tubular casts, glomerular degeneration, and tubular necrosis; these consequences of GNT administration were significantly preserved by the administration of EETT at doses of 300 and 450 mg/kg. The potential mechanism underlying the renal protective effects of *T. terrestris* on tubular damage, apoptosis, and oxidative stress in GNT-exposed rats may be attributed to its antioxidant properties [45]. This is likely due to the presence of flavonoids and saponins in *T. terrestris*, which have been shown to modulate various enzymes involved in cellular processes, such as division, proliferation, detoxification, inflammation, and immune response, according to Choi et al. [46].

This study observed a notable increase in serum creatinine, uric acid, and urea levels following GNT administration, although the elevation in serum creatinine was not deemed statistically significant. Notably, treatment with EETT, particularly at 450 mg/kg, demonstrated a substantial protective influence on serum levels of creatinine, urea, and uric acid, while this effect did not reach statistical significance.

This discrepancy may be attributed to the limited sensitivity of conventional renal biomarkers, which often fail to detect early injury. Their levels typically rise only after substantial nephron loss and are also affected by nonrenal variables, such as muscle mass, hydration status, and diet [47–49].

Alternatively, oxidative stress markers such as MDA, GSH, and CAT are capable of detecting early subclinical renal injury by reflecting shifts in intracellular redox balance. Several studies—including those on *Nigella sativa, Zingiber officinale, and Moringa oleifera*—have reported significant modulation of oxidative markers without corresponding changes in serum creatinine or urea levels [50–52]. For instance, the restoration of CAT and GSH activities was observed to precede measurable improvements in conventional renal parameters. Furthermore, emerging biomarkers such as neutrophil gelatinase–associated lipocalin (NGAL) and kidney injury molecule-1 (KIM-1) have demonstrated greater sensitivity in the early detection of AKI, often rising before elevations in traditional markers like creatinine and urea [53].

This study also indicated that the AETT did not exhibit significant nephroprotective effects, suggesting that the AETT has less ability to extract bioactive compounds. Conversely, EETT appears more effective in preserving bioactive compounds, particularly flavonoids and saponins known for their antioxidant activity.

These findings are consistent with previous research, demonstrating that solvent polarity plays a critical role in the extraction efficiency of phenolic and flavonoid compounds. For example, Do et al. reported that 100% ethanol extracts of *Limnophila aromatica* yielded the highest total phenolic and flavonoid content, along with enhanced antioxidant activity, compared to other solvents such as methanol and acetone [54]. Similarly, Mohammed et al. found that ethanol was more effective than other solvents in extracting flavonoids from a variety of plant materials [55]. Supporting this, Dailey and Vuong observed that absolute acetone and acetonitrile produced the lowest recovery yields of total phenolic compounds [56].

Furthermore, Bhebhe et al. corroborated these findings, emphasizing that the selection of extraction solvents significantly affects the recovery of phenolic content from plant materials [57, 58]. In agreement, a recent study on *T. terrestris* demonstrated that the antioxidant and enzyme-inhibitory activities of its extracts were strongly solvent-dependent, with acetone and ethanol showing the highest radical scavenging and reducing power [59].

To better contextualize our findings, many investigations have examined and compared *T. terrestris'* nephroprotective potential with that of other medicinal plants that are well-known for their kidney-protective abilities. *T. terrestris* showed notable protective effects against cisplatin-induced AKI in rats in a comparative study assessing the effects of standardized herbal decoctions defined in Ayurveda.

To further contextualize our findings, a comparative summary of previously reported nephroprotective effects of different medicinal plants against experimentally induced kidney injury in rodents was compiled (Table 2). This table highlights variations in plant species, nephrotoxic agents, dosing regimens, biomarkers, and overall outcomes. By aligning these reports with the present study on *Tribulus terrestris*, similarities and differences in nephroprotective responses become evident, thereby strengthening the interpretation of our results within the broader scope of phytomedicine research [60–70].

## 5. Conclusion

The study findings underscore the significant nephroprotective effect of an EETT against GNT-induced renal damage in normal rats. These benefits were supported by improvements in renal tissue damage, reduced levels of MDA, and increased levels of antioxidant enzymes, specifically CAT and GSH. Additionally, the higher dose of EETT at 450 mg/kg demonstrated more significant effects compared to the smaller doses of 150 and 300 mg/kg, indicating a potential dose-dependent relationship. Further research is required to investigate the dose–response curve of EETT and its impact on the treatment of GNT-induced kidney damage in rats. Conversely, various doses of AETT failed to demonstrate a protective effect against GNT-induced nephrotoxicity. Additional studies are recommended to elucidate the types and concentrations of phytochemical components of AEETT.

### 5.1. Limitation

There are multiple limitations to this study. First, a formal acute toxicity (LD_50_) evaluation of EETT was not conducted in the current study. Although the selected doses fall within the range commonly used in similar preclinical studies, and no signs of toxicity were observed in treated animals, this remains a limitation and should be addressed in future studies.

Second, the absence of genetic or inflammatory biomarker analyses restricted our ability to fully elucidate the mechanistic pathways underlying the observed nephroprotective effects. While oxidative stress markers were assessed, future studies incorporating proinflammatory cytokines and gene expression profiling could offer deeper mechanistic insights.

Finally, the 14-day treatment period may have been insufficient to capture long-term recovery or sustained effects of *T. terrestris* following AKI. Long-term studies are warranted to assess potential functional restoration and to evaluate any delayed therapeutic or adverse outcomes.

## Figures and Tables

**Table 1:** Phytochemical screening detected in EETT and AETT.

Phytochemical group	Detected in EETT	Detected in AETT
Saponins	+	−
Alkaloids	+	+
Flavonoids	+	−
Anthraquinones	+	−
Tannins	+	−
Terpenoids	−	+
Resins	−	+

*Note:* “+” indicates presence; “−” indicates absence based on qualitative phytochemical screening.

**Table 2:** Comparative summary of selected experimental studies on nephroprotective effects of herbal agents in rodent models.

Plant/extract	Nephrotoxic agent	Animal model/dose/route	Outcomes	Comparison with the current study	Reference
*Punica granatum* leaves, petroleum ether and methanol	Gentamicin (80 mg/kg i.m., for 8 days)	Rats/100, 200, 400 mg/kg/oral	Attenuated gentamicin-induced nephrotoxicity with significant reduction in serum creatinine, urea, TNF-α, and lipid peroxidation; restored antioxidant enzyme activities; improved renal histology.	EETT partially improved renal antioxidant status and histology at high doses, but serum creatinine and urea changes were mostly nonsignificant; AETT had no effect	[60]
*Safranal* (saffron active) 0.5 mL/kg/Pure compound	Gentamicin (80 mg/kg i.p., for 6 days)	Rats/0.5 mL/kg/i.p.	Significantly reduced BUN, creatinine, urinary glucose, and protein.	EETT demonstrated analogous antioxidant and nephroprotective effects, whereas AETT was less effective, with only high-dose EETT partially reducing uric acid and improving antioxidant enzymes.	[61]
*Tribulus terrestris* crude aqueous extract	Gentamicin (100 mg/kg i.p. for 5 days)	Rats/200 mg/kg/Oral	Treatment with *Tribulus terrestris* and vitamin C significantly improved renal function (BUN, creatinine, uric acid, β2M, NO, KIM-1), enhanced antioxidant status (CAT, SOD, GST, MDA), reduced histopathological damage, and decreased caspase-3 expression.	EETT at 450 mg/kg significantly improved histology and antioxidant enzymes (GSH, CAT), partially reduced MDA, and lowered uric acid levels nonsignificantly; AETT showed no meaningful effect	[62]
*Punica granatum* (fruit extracts) petroleum ether, chloroform, and methanol extraction.	Gentamicin (100 mg/kg i.p. for 8 days)	Rats/100, 200, 400 mg/kg/oral	Reduced serum/urine creatinine, urea, uric acid; increased SOD, CAT, GSH; decreased lipid peroxidation; dose-dependent histological improvement.	EETT exhibits similar antioxidative and nephroprotective, though dose dependence was more pronounced in the present work.	[63]
*Rheum turkestanicum* (roots) 70% ethanol	Gentamicin (80 mg/kg i.p., for 6 days)	Rats/100, 200 mg/kg/i.p.	Reduced serum creatinine, BUN, MDA; increased thiol levels; improved renal histopathology.	EETT at high dose showed histological restoration and improved antioxidant enzymes; serum creatinine and urea changes were not statistically significant.	[64]
*Zingerone* (from ginger) pure compound	Gentamicin (100 mg/kg i.p. from Day 8 to Day 14)	Rats/10 mg/kg/oral	Reduced Cr, BUN, NGAL, KIM-1; decreased MDA, PC, NO; enhanced SOD, CAT, GPx, GSH; histological protection.	EETT mirrored antioxidant improvements at high doses and partially improved histology; serum Cr and BUN changes nonsignificant; AETT had no effect	[65]
*Mucuna pruriens* (leaves) ethanolic extract	CCl4 (3 mL/kg bw i.p.) or rifampicin (250 mg/kg bw oral) single dose	Rats/50, 100 mg/kg/oral	Reversed elevated urea, uric acid, bilirubin; restored antioxidants; histology improved.	EETT partially restored renal antioxidant enzymes and histology at high dose; serum urea and creatinine mostly unchanged; AETT ineffective	[66]
*Boerhavia diffusa* root aqueous extract	Gentamicin (150 mg/kg/day, for 10 days)	Rats/200, 400 mg/kg/oral	Reduced BUN, creatinine, MDA; increased GSH; histology preserved; comparable to α-lipoic acid.	High-dose EETT reduced MDA, improved GSH/CAT, and restored histology; serum biochemical markers nonsignificant.	[67]
*Tribulus terrestris* (fruit) 70% ethanol	Cisplatin (5.5 mg/kg) i.p. single dose)	Mice/100, 300, 500 mg/kg/oral	Prevented decreases in body/kidney weight; reduced tubular dilatation and congestion; dose-dependent recovery.	EETT at high doses similarly improved histology and antioxidant status.	[68]
*Olea europaea* (leaves) ethanolic extract	Cisplatin (6 mg/kg i.p.) single dose	Rats/150, 300 mg/kg/oral/	Reduced Cr, BUN; increased Cr clearance and potassium; dose-dependent histological improvement.	EETT restored renal tissue and antioxidant enzymes at high dose; serum Cr and BUN changes nonsignificant.	[69]
*Eurycoma longifolia* (root) water extract	Paracetamol (200 mg/kg p.o., for 14 days)	Rats/100, 200, 400 mg/kg/oral	Dose-dependent: Decreased serum creatinine and urea; increased Cr clearance, albumin, total protein; histology preserved.	EETT at high dose improved histology and antioxidant enzymes; serum creatinine and urea change nonsignificant; AETT showed no effect	[70]

*Note:* This table presents representative studies on the nephroprotective effects of medicinal plants against chemically induced kidney injury in rodents. It highlights variations in plant species, nephrotoxic agents, dosing regimens, and biomarkers, in comparison with the present *Tribulus terrestris* study. CAT: catalase; Cr: creatinine; i.m.: intramuscular; i.p.: intraperitoneal; p.o: GPx: glutathione peroxidase; GSH: reduced glutathione; LPO: lipid peroxidation; MDA: malondialdehyde.

Abbreviations: BUN, blood urea nitrogen; KIM-1, kidney injury molecule-1; NGAL, neutrophil gelatinase–associated lipocalin; NO, nitric oxide; PC, protein carbonyls; SOD, superoxide dismutase; TNF-α, tumor necrosis factor alpha.

## Data Availability

All data supporting the findings of this study are available from the corresponding author upon reasonable request.
